# Advancing Fault Detection in HVAC Systems: Unifying Gramian Angular Field and 2D Deep Convolutional Neural Networks for Enhanced Performance

**DOI:** 10.3390/s23187690

**Published:** 2023-09-06

**Authors:** Wunna Tun, Kwok-Wai (Johnny) Wong, Sai-Ho Ling

**Affiliations:** 1 Faculty of Design, Architecture and Building, University of Technology Sydney, Ultimo, NSW 2007, Australia; wunna.tun@student.uts.edu.au (W.T.); johnny.wong@uts.edu.au (K.-W.W.); 2 Faculty of Engineering and IT, University of Technology Sydney, Ultimo, NSW 2007, Australia

**Keywords:** heating, ventilation and air conditioning (HVAC), Gramian angular field (GAF), convolutional neural networks (CNNs), fault detection and diganosis (FDD), HVAC SIMulation PLUS (HVACSIM+)

## Abstract

Efficiency and comfort in buildings rely on on well-functioning HVAC systems. However, system faults can compromise performance. Modern data-driven fault detection methods, considering diverse techniques, encounter challenges in understanding intricate interactions and adapting to dynamic conditions present in HVAC systems during occupancy periods. Implementing fault detection during active operation, which aligns with real-world scenarios and captures dynamic interactions and environmental changes, is considered highly valuable. To address this, utilizing the dynamic simulation system HVAC SIMulation PLUS (HVACSIM+), an HVAC fault model was developed using 194 sensor signals from each HVAC component within a single-story, four-room building. The advanced HVAC fault detection framework, leveraging simulated HVAC operational scenarios with the Gramian angular field (GAF) and two-dimensional convolutional neural networks (GAF-2DCNNs), offers a robust and proactive solution. By utilizing the GAF capacity to convert time-series sensor data into informative 2D images, integrated with 2DCNN for automated feature extraction, hidden temporal relationships within 1D signals are captured. After training on nine significant HVAC faults and normal conditions during occupancy, the effectiveness of the proposed GAF-2DCNN is evaluated through comparisons with support vector machine (SVM), random forest (RF), and hybrid RF-SVM, one-dimensional convolutional neural networks (1D-CNNs). The results demonstrates an impressive overall accuracy of 97%, accompanied by precision, recall, and F1 scores that surpass 90% for individual HVAC faults. Through the introduction of the unified approach that integrates HVACSIM+ simulated data and GAF-2DCNN, a notable enhancement in robustness and reliability for handling substantial HVAC faults is achieved.

## 1. Introduction

Modern building efficiency and occupant comfort are intricately tied to the optimal operation of heating, ventilation, and air conditioning (HVAC) system. These systems play an important role in maintaining desired indoor conditions, impacting factors such as occupant comfort and energy consumption. Underscoring the significance of identifying operational faults is the fact that undetected issues can result in energy inefficiencies. The timely detection and diagnosis of HVAC faults can mitigate energy wastage and prevent complete equipment breakdown [1]. In line with this, numerous fault detection and diagnosis (FDD) strategies have been developed to address energy-saving issues, with the aim of enhancing system efficiency [2]. Extensive research [3,4] has been investigated into optimizing air handling units (AHUs) control strategies to achieve better operational efficiency. However, AHU controllers are susceptible to various faults, including sensor reading inaccuracies and control errors, signal line failures, and blockages in dampers and hot water valves, all of which compromise energy efficiency and result in costs [5]. Therefore, effective HVAC equipment fault detection is essential to ensure timely maintenance or replacement.

The evolution of HVAC fault detection has transitioned towards data-driven approaches, offering a diverse techniques to enhance system monitoring and diagnosis. In the earlier stages, rule-based fault detection approaches were introduced, specifically tailored for air handling units (AHUs) within HVAC systems [6,7,8]. These approaches rely on predefined conditions and thresholds to identify anomalies and deviations from normal system behavior. Nevertheless, a significant limitation of rule-based methods lies in their dependence on predefined rules and thresholds. Moreover, as HVAC systems operate under diverse conditions and occupant patterns, these methods may encounter difficulties in adapting to dynamic scenarios, load variations, and intricate system behaviors. Consequently, the potential for such rule-based systems to generate false positives is elevated due to the inflexible nature of the predetermined rules.

Recent advancements in artificial intelligence (AI) and machine learning (ML) have opened the door for the development of automated HVAC fault diagnostic systems. A data-driven approach was proposed for HVAC chiller systems using principal component analysis (PCA) to identify anomalies and a reconstruction-based contribution method to identify fault-related variables [9]. Notably, its primary strength lies in its capability to function effectively even without prior knowledge or historical data concerning unforeseen occurrences. However, challenges might arise in identifying complex fault-related factors, and there could be challenges related to the scalability and adaptability of the decision table approach. Another study introduced a diagnostic bayesian network framework [10], leveraging probabilistic modeling to capture complex variable relationships and enhance fault understanding. Nevertheless, its complexity, reliance on expert knowledge, and applicability to specific equipment can present limitations.

With continuous advancements in statistical machine learning and information theory, fault detection, and classification in HVAC systems using artificial neural networks (ANNs) [11,12], general regression neural networks (GRNN) [13], which are wavelet-based neural networks [14], have become increasingly essential. The studies focus on the practical application of HVAC fault detection, employing artificial neural networks (ANNs) renowned for effectively handling complex data relationships. However, potential challenges could arise from the complexity of feature extraction, selection, and scaling from the raw sensor data, as well as in generalizing the findings to diverse HVAC systems. The extraction and selection of irrelevant features and improper scaling can adversely affect the learning process, leading to biased outcomes and slower convergence. Therefore, it is essential to ensure appropriate feature extraction and scaling to achieve optimal performance when utilizing ANNs for HVAC fault detection and classification.

An intelligent swarm-based artificial neural network (ANN) model, augmented with the ensemble rapid centroid evaluation (ERCE) technique [15], is introduced. This approach effectively selects important features by leveraging the relative entropy between low- and high-frequency features. The ASHRAE-1312-RP dataset, reflecting diverse HVAC fault types and behaviors, is employed for experimentation. The selected features show reduced redundancies and enhanced model performance compared to manual selection. However, a notable limitation arises due to the lack of simulated data for generalization evaluation, which can potentially give insights into the model adaptability to various HVAC operating conditions. Importantly, the consequences of inaccurate or inadequate feature selection could compromise the entire fault detection and classification process.

In [16], a novel decentralized Boltzmann-machine-based approach for HVAC air handling unit (AHU) fault diagnosis is introduced. It tackles challenges associated with correlated fault indicators and computational demands by utilizing less affected residuals as indicators and employing a unique decentralized voting mechanism for effective sensor fault localization. While the method exhibits high accuracy in diagnosing sensor faults, it is constrained by potential reliance on residual data quality. The experiments use ASHRAE Project 1312-RP data, comparing AHU-A with faults to AHU-B under normal conditions across various seasons. However, the study lacks HVAC operational simulated data, which is advantageous for comprehensive testing and validation of fault detection and diagnosis methodologies, potentially limiting performance insights into across diverse operating conditions and fault scenarios.

In the context of enhancing building energy conservation, the utilization of a deep belief network for detecting various HVAC faults in air-conditioning systems is introduced [17,18]. Despite the strength of its layer-wise training for learning intricate patterns, the method may struggle to effectively capture the spatial and temporal relationships inherent in HVAC data. Moreover, the focus on only five faults in the paper might not comprehensively represent the reality, where there could be more diverse faults arising from human errors, unexpected device malfunctions, and sensor drift. This highlights the need for further exploration of a broader range of AHU faults to ensure the diagnostic model accuracy.

Recent attention has been drawn to one-dimensional convolutional neural networks (1D-CNNs) in analyzing raw sensor time series signals due to its robust classification performance, automatic feature extraction, and computational efficiency [19,20]. The effectiveness of the proposed method is verified through experimentation with a fault dataset derived from a typical building HVAC systems (chiller) within the ASHRAE research project 1043 (RP-1043). While this method presents strengths in providing better accuracy, it should be noted that it is limited in its use of simulation data for testing model generalization. Furthermore, the constraints of employing a 1D-CNN become more apparent when dealing with multiple HVAC fault classifications, as its focus on temporal patterns might hinder its ability to capture complex spatial interdependencies within HVAC systems.

To overcome this challenge, emerging techniques such as Gramian angular fields (GAF) transform time-series data into spatial representations, with recent advancements integrating algorithms with CNNs to enhance fault diagnosis capabilities [21,22,23]. In [24], the GAF-2DCNNs approach focuses on enhancing the deep learning application for HVAC fault detection system is proposed. It involves using pruning to significantly decrease model parameters and incorporating layer-wise relevance propagation (LRP) for improving model interpretability. To assess its effectiveness, 31 faults data simulated from real HVAC systems in Japan are employed. The results demonstrate a classification accuracy of 90%, while also reducing the dimensions of model by over 99%. However, focusing only on 47 AHU operational parameters might overlook some significant HVAC system behaviors, while requiring additonial steps, such as pruning and LRP for 24-h cycle training. In practice, it is important to evaluate the effectiveness of the system in various HVAC faults and normal situations during occupied period, as false positives and negatives could result in unnecessary alarms. Additionally, the generalizability can be assessed by comparing it with other machine learning-based FDD systems and utilizing various benchmark datasets.

Overcoming the above challenges, the study makes a significant contribution to the field of HVAC fault detection by introducing unified approach combining HVACSIM+ simulated data and transformative GAF-2DCNNs. By integrating the Gramian angular field (GAF) and two-dimensional convolutional neural networks (2D-CNNs), the proposed approach leverages on the spatial insights offered by GAF representations and the feature extraction capabilities inherent in 2D-CNN, leading to enhanced identification of HVAC faults. The seamless integration of HVACSIM+ simulated data within the proposed FDD framework further strengthens its applicability. By combining real-world complexities with simulated operational scenarios, the study offers a comprehensive understanding of HVAC system behavior. This integration enriches the fault model and broadens the scope of insights, ensuring that the proposed methodologies are robust and reliable under various operational conditions. Below is a summary of the substantial contributions made by this study:

- Leveraging Simulated Data from HVACSIM+: This utilizes the dynamic simulation system HVAC SIMulation PLUS (HVACSIM+) to simulate HVAC faults from 194 sensor signals within a single-story, four-room building (each measuring 400 m^2^), offering precise control over operational conditions and encompassing diverse faults. This approach enhances the accuracy and applicability of fault detection models, resulting in improved performance and more effective real-world implementations in HVAC systems. This distinct dataset source enriches the originality of our approach and reinforces its applicability to real-world HVAC scenarios.
- Normal State Inclusion and Multi-Fault Classification: The study further extends its contributions by training the GAF-2DCNNs model on nine significant HVAC faults and normal conditions. Incorporating normal conditions in a FDD system offers advantages such as reducing false alarms, improving accuracy, and enhancing system performance. The evaluation of the proposed system was conducted using precision, recall, and F1 score metrics, revealing significant performance for each detected fault.
- Strategic Training During Operational Hours Enhances Robustness: Aligning with real-world HVAC systems conditions, training during occupied periods from 6 AM to 6 PM offers significant advantages. This approach captures specific usage patterns, behaviors, and anomalies that are more likely to arise during these times. Consequently, the model gains insights into dynamic system behavior, load fluctuations, and environmental influences associated with building occupancy, leading to robust and precise fault detection in real-world contexts.
- Enhanced Time Resolution: By employing a finer time resolution of 1 min in HVAC fault detection, this study achieves enhanced accuracy by capturing intricate variations within shorter intervals, thereby improving system behavior analysis and anomaly detection. In contrast to a larger sample size like 15 min, the utilization of 1-min intervals offers the advantage of capturing more frequent data points. This approach allows for a more detailed comprehension of system dynamics, enabling the identification of rapid changes and transient patterns that may go unnoticed with larger intervals. Consequently, the finer time resolution significantly elevates the precision and sensitivity of the fault detection process.
- Occupancy-Aware Modeling: The implementation of occupancy-aware modeling is another notable contribution of this study. By focusing on HVAC fault detection during occupied periods, the model captures specific usage patterns and behaviors that are relevant to real-world HVAC systems conditions. Its tailored approach enhances the ability of the model to distinguish between normal and faulty operations, providing more accurate results and enabling quick decision making.
- Rigorous Validation Against Established Benchmarks: The validation against established benchmarks, including ASHRAE data, enhances credibility and reliability of the proposed model. This validation process aligns with industry standards, boosting confidence in the effectiveness and practicality of the proposed methodologies. By benchmarking against reputable references, the study establishes a solid foundation for evaluating the performance of the proposed unified approach combining HVACSIM+ simulated data and GAF-2DCNNs.
- Evaluating Model Effectiveness: A comprehensive comparison with support vector machine (SVM), random forest (RF), and hybrid RF-SVM, one-dimensional convolutional neural networks (1D-CNNs), is carried out to explore the effectiveness of the proposed GAF-2DCNNs model. The noticeable result shows the superiority of the GAF-2DCNN approach in accurately identifying nine significant HVAC faults and fault-free scenarios within simulated operational dynamics. An added advantage of considering fault detection during occupancy is the reduction of model computation time, which enhances the practicality of real-time fault detection and quick decision making without the need for extensive computation resource. This feature makes the approach more feasible for applications that require timely responses.

In summary, the incorporation of advanced GAF-2DCNNs techniques, seamless integration of HVACSIM+ simulated data, occupancy-aware modeling, capturing finer time resolution of 1 min, and rigorous validation against benchmarks ASHRAE data collectively contribute to the advancement of HVAC fault detection. The utilization of GAF and 2D-CNN methodologies, along with the innovative approach to address operational complexities, makes this study a significant step forward in enhancing HVAC fault detection system. Despite system complexity, the unified approach combining HVACSIM+ simulated data and GAF-2DCNNs consistently achieves better accuracy, precision, recall, and F1 scores. This approach effectively reduces false positives and enhances fault detection, showcasing its robustness and reliability in addressing major HVAC fault scenarios.

The upcoming Section 2 provides insight into the HVAC Simulation PLUS (HVACSIM+) parameters and the simulation process applied to a single-story building with nine distinct types of HVAC faults and normal condition. The theoretical foundation of transforming time series into images using the GAF and the structure of the 2D-CNN are covered in Section 3.1 and Section 3.2, respectively. The evaluation and discussion of the proposed GAF-2DCNNs fault detection system are presented in Section 5. Lastly, Section 6 gives the limitations, conclusions, and potential future directions of the proposed unified framework.

## 2. HVAC Faults Simulation Using HVACSIM+

**Figure 1:**
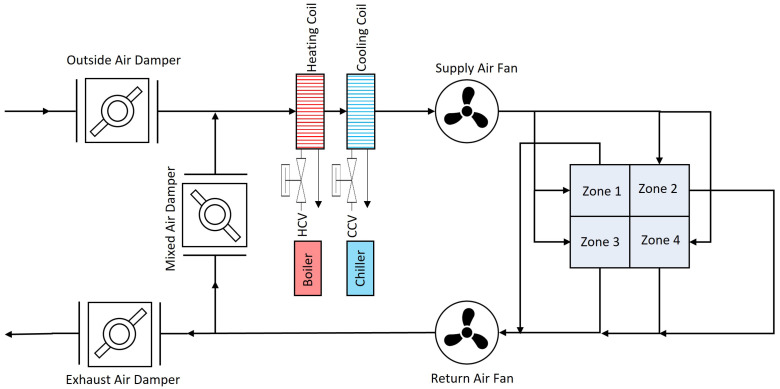
Schematic representation of HVAC systems.

To enhance the fault detection and diagnosis of the HVAC systems, this study utilized the HVACSIM+ simulation model [25] to generate both normal and faulty operational data. The simulation was carried out for a single-story, four-room building (each measuring 400 m${}^{2}$), ensuring exposure to external heat loads across the rooms. The air conditioning of the building was provided through an air-handling unit (AHU) system with four zones, each featuring a variable air volume fan to regulate zone temperatures. The HVAC system layout and energy mode setup are given in Figure 1. The AHU system incorporated essential components, such as preheating coils, cooling coils, heating coils, heating and cooling coil control valves, outside air dampers, and conditioned air outlets. A duct facilitated air circulation between the AHU and the rooms. The simulation model was equipped with sensors to continually monitor the pressure, temperature, humidity, and airflow characteristics of the system.

### 2.1. HVAC Control Algorithm Pseudocode

The implementation of the HVAC systems simulated model aligns with the provided pseudocode Algorithm 1, which outlines the HVAC control algorithm based on the specified inputs and desired operating conditions. It involves a series of steps to manage the room temperature and occupancy while considering various parameters. The initial inputs include the desired temperature for the zone, maximum room capacity, temperature tolerance range, cooling power, and heating power of the HVAC systems. The algorithm begins by retrieving the current temperature and occupancy status. Subsequently, the temperature control process is carried out, following a series of conditional statements. If the current temperature exceeds the desired range plus the specified tolerance, the algorithm calculates the cooling power required and applies it to the room. Conversely, if the current temperature falls below the desired range minus the tolerance, the algorithm calculates the heating power required and applies it. In cases where the current temperature falls within the acceptable range, the algorithm maintains the temperature at its current level. The pseudocode (Algorithm 1) given in this study works as a guide for executing the HVAC control algorithm and managing temperature and occupancy dynamics within the room.
**Algorithm 1** HVAC Control Algorithm Pseudocode**Require:** Inputs:
  1:ZONE_TEMPERATURE: Set Desired Temperature  2:ROOM_CAPACITY: Maximum Occupancy  3:TOLERANCE: Temperature Tolerance Range  4:COOLING_POWER: HVAC Systems Cooling power  5:HEATING_POWER: HVAC Systems Heating power  6:   7:**function** RetrieveCurrentTemperatureAndOccupancy  8:    current_Temperature← getCurrentTemperature()  9:    current_Occupancy← getCurrentOccupancy() 10:**end function** 11:  12:**function** TemperatureControl 13:    **if** current_Temperature>(ZONE_TEMPERATURE+TOLERANCE) **then** 14:        **Calculate cooling power** 15:     COOLING_POWER←(current_Temperature−ZONE_TEMPERATURE) 16:     ×SET_COOLING_POWER 17:        Apply cooling power 18:    **else if** current_Temperature<(ZONE_TEMPERATURE−TOLERANCE) **then** 19:        **Calculate heating power** 20:     HEATING_POWER←(ZONE_TEMPERATURE−current_Temperature) 21:     ×SET_HEATING_POWER 22:        Apply heating power 23:    **else** 24:        **Maintain temperature** 25:    **end if** 26:**end function**


### 2.2. Design Parameter Consideration

The HVACSIM+ simulated model, as presented in Figure 1, comprises the AHU with essential components, such as air supply and exhaust fans, preheating coils, cooling coils, heating coils, control valves for heating and cooling coils, external air dampers, and conditioned air outlets. The operating characteristics of HVAC systems are continuously monitored through pressure, temperature, humidity, and airflow sensors, following the steps outlined in Algorithm 1. Additionally, a variable air volume (VAV) system regulates zone temperatures, channeling conditioned air to terminal units and zones via a duct network. Each terminal unit incorporates a modulating damper controlled by a thermostat, responding to cooling requirements within the zone. Employing diverse controllers for supply air temperature, fan speed, and room temperature, the VAV system achieves the desired zone temperature set point. When the room temperature ($T_{ZA}$) varies from the set point ($T_{set,ZA}$), the controller activates, adjusting the internal VAV box damper to regulate airflow volume and maintain comfortable indoor thermal conditions. Notably, Table 1 outlines key specifications for building and HVAC design.

However, as the VAV box damper undergoes repeated modulations, it introduces alterations in the static pressure within the supply air duct. Subsequently, the supply fan controller responds by adjusting the speed of the supply fan, taking into account the discrepancy between the static pressure within the duct ($P_{SA}$) and the predefined static pressure set point within the duct ($P_{set,SA}$). This process aims to effectively regulate the supply air temperature ($T_{SA}$) of the AHU in the VAV system. To achieve this, the cooling coil valve controller precisely modulates the cooling coil valve (CCV), thereby regulating the flow rate of cooling coil water. This adjustment is guided by the difference between the supply air temperature ($T_{SA}$) and the specified supply air temperature set point ($T_{set,SA}$).

Utilizing the above control method ensures the stable operation of the VAV system. The simulation model is subjected to a 24-h test, during which data are gathered at 1-min intervals for each sensor reading. By incorporating 194 sensor readings into the HVAC system analysis, this approach offers distinct advantages compared to relying solely on the 47 AHU operational parameters [24]. Moreover, increasing the frequency of sensor data sampling to every 1-min interval provides a more complete understanding of system behavior. This approach captures subtle changes and interconnections that could be missed when using a limited parameter set sampled at 15-min intervals. The finer granularity not only enhances the precision of fault detection, but also ensures a more accurate representation of real-world operational scenarios. This holistic approach, embracing a wider range of sensor data, ultimately strengthens fault detection accuracy, early anomaly identification, and the practical applicability of the HVAC system analysis.

In accordance with Algorithm 1, the HVAC simulation model developed in this study was adapted for summer conditions. These scenarios were broken down into AHU settings, zone configurations, and adjustments to the heating and cooling systems. The simulations extended over 24-h periods, with occupancy scheduled from 6:00 AM to 6:00 PM. By emphasizing occupancy periods, a practical understanding of HVAC system behavior is achieved, enhancing the relevance and applicability of the findings. The approach sets this study apart from [24], highlighting the benefits of exclusively considering occupancy periods in HVAC system analysis.

Furthermore, a minimum outdoor air damper opening of 40% was implemented to ensure ventilation. The economizer control came into play when the outdoor air temperature dropped below 18 ${}^{\circ }$C, complemented by a supply air temperature of 12.77 ${}^{\circ }$C. The fan speeds were managed to maintain duct pressure, while the return fan operation was synchronized to 80% of the supply fan speed. The target room temperature during occupied hours was set at 21 ${}^{\circ }$C, and airflow was adjusted, ranging from 200 to 1000 cfm across different zones. The study considered three normal days and nine significant faults, involving minute-by-minute data sampling from 194 sensors, culminating in 1440 samples over a 24-h period.

### 2.3. Validating HVACSIM+ Simulation with ASHRAE Data

**Figure 2:**
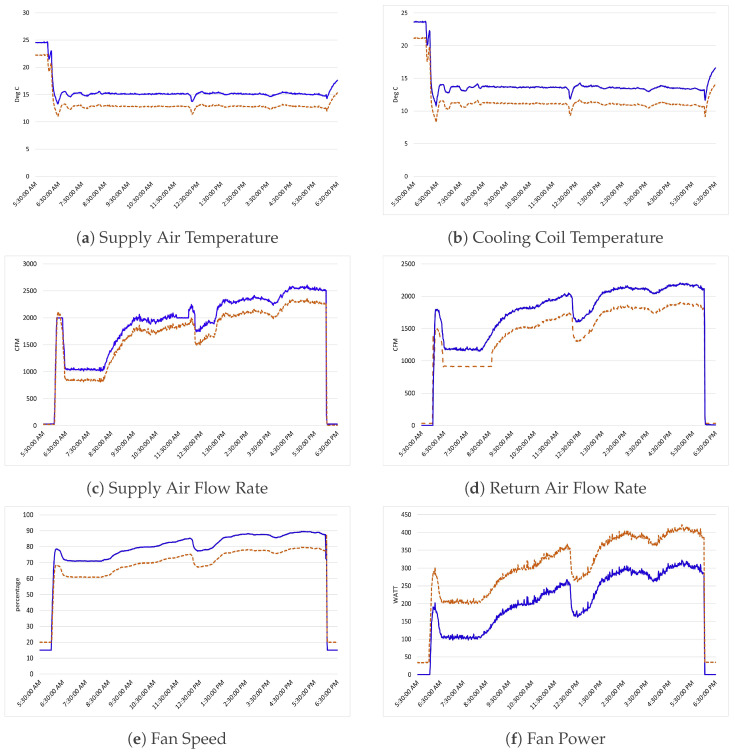
Comparison analysis of simulated and ASHRAE HVAC operation data [26]. (**‒‒‒‒** HVACSIM+ Simulated Data; **_ _ _ _** ASHRAE Data).

The study incorporates a comprehensive validation process, which compares the dynamic behavior of HVAC systems parameters, such as time series data and fault scenarios, produced by the HVACSIM+ simulation model with benchmark ASHRAE experimental data [26]. To establish this validation, a direct comparison has been made between the simulation-generated time series data and fault scenarios, and the real-world data sourced from ASHRAE, as given in Figure 2. For validation purposes, the focus was directed towards significant parameters: supply air and cooling coil temperature, supply air and return air flow rate, fan speed, and power consumption.

A comparison between simulated supply air and cooling coil temperature and corresponding ASHRAE experimental data, as given in Figure 2a,b, revealed a striking resemblance in their temporal fluctuations. The observed peaks and troughs closely aligned in both datasets, signifying accurate capture of temperature dynamics by the simulation model. The similar trends shown when examining air flow rate data, as given in Figure 2c,d, as both simulated and experimental records displayed consistent oscillations in air flow rate, especially during system adjustments or external influences. The similarity strengthens confidence in the accuracy of the HVACSIM+ simulation when showing how air flows within the system.

Additionally, upon examining the fan speed data, as in Figure 2e, a strong similarity emerges between the simulation and ASHRAE measurements. The simulation effectively reproduces how fan speed reacts to changes in system load and operational conditions, thus reinforcing its fan modeling accuracy. Moreover, Figure 2f indicates that the simulation of fan power consumption closely resembles the actual power consumption data collected by ASHRAE. The consistency in power consumption trends supports the ability of HVACSIM+ simulation to offer dependable estimates of power needs. The alignment between the simulation and ASHRAE data during validation is especially promising. It not only proves that the simulation is accurate, but also gives more confidence to use it for HVAC faults model for classification. While the HVAC systems simulation involves a total of 194 parameters, the comparison studies were conducted specifically on key parameters, such as supply air temperature, cooling coil temperature, supply air flow rate, return air flow rate, fan speed, and fan power, with illustrative examples given in Figure 2a–f.

### 2.4. Brief Description of Significant Faults

The selection of specific faults in the HVAC system analysis is grounded in their practical relevance and potential impact on system performance. The HVACSIM+ simulation model employed in this study considered a wide range of significant faults that are particularly complex and difficult to handle effectively, such as the “cooling coil valve fully opened” fault (CCV100%OP), where the valve is manually set to open completely. This activation prompts the heating coil to regulate supply air temperature, sometimes coupled with an unintended control strategy fault that closes the outdoor air damper during heating coil operation. Similarly, the “cooling coil valve 100% closed” fault (CCV100%CL) can lead to excessive cooling or inadequate temperature regulation, which results in deviations in supply air temperature, opening zone VAV dampers, increasing supply airflow, and increasing fan speeds. The simulation model also investigates the “cooling coil valve reverse action” fault (CCVREV). The consideration of the CCVREV is important as it represents a scenario where expected system behavior is intentionally reversed, often resulting from control system or actuator faults. In addition, it allows to assess the effectiveness of fault detection in identifying such complex deviations, enhancing its adaptability and applicability to real-world HVAC systems.

Furthermore, the “duct leak after the supply air fan” (DLAFTSF) fault is simulated, impacting duct flow resistance and subsequently affecting air pressure, airflow, and room temperature. Incorporating the “stuck exhaust or outside air dampers” (EADAMPOP and EADAMPCL) faults into the model is significant because these faults directly impact the ventilation and air circulation within HVAC systems. These faults can lead to compromised indoor air quality, energy inefficiency, and improper temperature regulation. Lastly, the “outside air damper 45% opened” (OADAMP45%OP) and “outside air damper closed” (OADAMPCL) faults are examined, affecting the proportion of recirculated air due to simulated stuck damper positions. The validation process entails a thorough comparison between simulated and real-world data across critical parameters, ultimately enhancing the model accuracy and applicability across various contexts.

In the process of implementation, data were extracted specifically from the occupancy timeframe, resulting in 720 data points over a 12-h span (6 AM–6 PM). Outside of this active period, sensor readings exhibited minimal fluctuations as clearly presented in Figure 2, rendering them unsuitable for effectively modeling decision-making patterns. In the pursuit of generating a robust model, a compilation of 2160 normal samples was gathered across three fault-free days. Additionally, for each distinct fault type outlined in Table 2, 720 fault samples were collected. This targeted strategy during occupy period avoids additional steps, such as pruning and LRP, which would otherwise be needed to sort through less informative nighttime data. This focused technique not only enhances accuracy but also streamlines the architecture of the model, maintaining efficiency without the added complexity of 24-h training [24]. This methodology fits well with HVAC fault detection needs, ensuring accuracy, simplicity, and efficiency.

## 3. Advanced HVAC Fault Detection System

**Figure 3:**
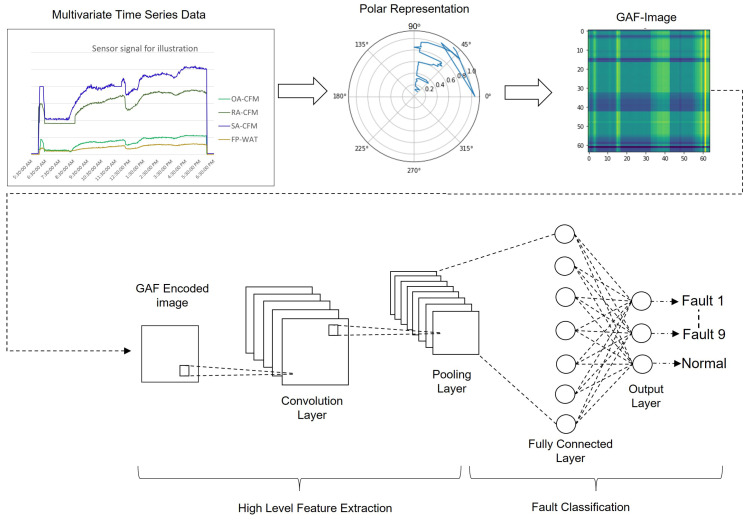
The HVAC fault detection system using GAF-2DCNNs.

This section introduces a data-driven HVAC fault detection approach employing Gramian Angular Fields (GAF) and two-dimensional convolutional neural networks (CNNs). As given in Figure 3, the GAF technique encodes HVAC sensor time series into images (Section 3.1), subsequently processed by 2DCNN for fault classification (Section 3.2). The GAF-2DCNNs integrated framework begins by encoding simulated time series data into 2D GAF images. This transformation translates temporal changes into spatial patterns, enabling the CNN to capture spatial relationships and patterns that might carry essential fault-related information. In addition, the encoded GAF images work as a bridge between temporal dynamics and spatial patterns, enhancing the ability of the model to detect latent fault signatures.

During the training of the 2D-CNN, it iteratively learns from these images that represent various HVAC systems conditions, both normal and faulty conditions. It adjusts and optimizes the network parameters to identify slight changes in patterns related to different HVAC systems states. The efficiency of the proposed unified framework, trained using HVACSIM+ simulated data outlined in Table 2, is validated through benchmark ASHRAE data [26], with comparison studies against Support Vector Machine (SVM) [27,28], Random Forest (RF) [29], and hybrid HVAC systems fault diagnosis models [30] to show the superior accuracy of the proposed GAF-2DCNNs approach. Notably, these methods require feature extraction and selection, whereas the proposed approach stands out for its superior accuracy. Moreover, it is important to validate the proposed fault detection system in comparison to the one-dimensional CNNs (1DCNNs) [20], highlighting its ability to capture both temporal and spatial information, which is a feature not captured by the 1DCNNs approach.

### 3.1. Gramian Angular Field

The Gramian Angular Field (GAF) is a unique technique used in time series analysis. It converts time series data into images by encoding temporal relationships and correlations onto a two-dimensional polar coordinate system [31]. It transforms data points into angles and distances, with cosine values representing the angular differences. The resulting image captures complex temporal patterns, making it valuable for tasks such as fault detection and signal processing. For the given time series $X=\{x_{1},x_{2},\ldots ,x_{n}\}$, the first step in GAF is to normalize it into values interval of [0,1] by:

(1) $${\tilde{x}}_{i}^{}=\frac{\left(x_{i}-\max\left(X\right)+x_{i}-\min\left(X\right)\right)}{\max(X)-\min(X)}$$

(2) $${\tilde{x}}_{i}^{}=\frac{x_{i}-\min\left(X\right)}{\max(X)-\min(X)}$$

After normalization, the normalized time series data are represented in the polar coordinate system by encoding the value as the angular cosine and the time stamp as the radius with the equation below:

(3) $$\phi_{i}=\arccos\left({\tilde{x}}_{i}^{}\right),-1\leq {\tilde{x}}_{i}^{}\leq 1,{\tilde{x}}_{i}^{}\in {\tilde{X}}_{}^{}$$

(4) $$r_{i}=\frac{t_{i}}{N},t_{i}\in N$$

where ϕ∈[0,π], $t_{i}$ is the time stamp, and *N* is a constant factor to regularize the span of polar coordinate. The GAF has essential properties of rescaling time series data into different intervals with different angular bounds, whereas [0,1] corresponds to the cosine function in $\left[0,\frac{\pi }{2}\right]$, while cosine values in the interval [−1,1] fall into the angular bounds [0,π]. After transforming the re-scaled time series to a polar coordinate system, we can identify temporal correlations within different time intervals considering the trigonometric sum or difference between each point. GAF can generate two images by different equations. The Gramian Summation Angular Field (GASF) is defined in Equations (5) and (6) and the Gramian Difference Angular Field (GADF) is defined in Equations (7) and (8), which allow easy calculation of the angular viewpoint, where (GASF) is based on cosine functions and (GADF) is based on sine functions:

(5) $$\mathrm{GASF}=\left[\begin{array}{ccc} \cos\left(\phi_{1}+\phi_{1}\right) & \cdots & \cos\left(\phi_{1}+\phi_{n}\right)\\ \cos\left(\phi_{2}+\phi_{1}\right) & \cdots & \cos\left(\phi_{2}+\phi_{n}\right)\\ \vdots & \ddots & \vdots \\ \cos\left(\phi_{n}+\phi_{1}\right) & \cdots & \cos\left(\phi_{n}+\phi_{n}\right) \end{array}\right]$$

(6) $$\begin{array}{cc} GASF & ={\tilde{X}}^{\prime }\cdot \tilde{X}-{\sqrt{I-{\tilde{X}}^{2}}}^{\prime }\cdot \sqrt{I-{\tilde{X}}^{2}} \end{array}$$

(7) $$\mathrm{GADF}=\left[\begin{array}{ccc} \sin\left(\phi_{1}+\phi_{1}\right) & \cdots & \sin\left(\phi_{1}+\phi_{n}\right)\\ \sin\left(\phi_{2}+\phi_{1}\right) & \cdots & \sin\left(\phi_{2}+\phi_{n}\right)\\ \vdots & \ddots & \vdots \\ \sin\left(\phi_{n}+\phi_{1}\right) & \cdots & \sin\left(\phi_{n}+\phi_{n}\right) \end{array}\right]$$

(8) $$\begin{array}{cc} GADF & ={\sqrt{I-{\tilde{X}}^{2}}}^{\prime }\cdot \tilde{X}-{\tilde{X}}^{\prime }\cdot \sqrt{I-{\tilde{X}}^{2}} \end{array}$$

where *I* is the unit row vector. The GAF algorithm has several advantages, as it considers maintaining temporal dependency as per-position movement throughout the time period. This means it can quickly convert a one-dimensional time series into a two-dimensional image that can be effectively used by a two-dimensional deep convolutional neural networks, as described in Section 3.2.

### 3.2. Deep Convolution Neural Network

This section outlines the fundamental algorithms and structures of the deep convolutional neural networks (CNNs) [23], as well as its training methodology, utilized for classifying time series images transformed through GAF in Section 3.1. Essentially, the architecture of CNN consists of two main parts: the initial part utilizes convolution and pooling operations to generate a feature map from the raw input signal, employing an appropriately chosen kernel size. The subsequent part focuses on classifying the most intricate features, collaborating with a multi-layer perception (MLP) method. Figure 3 illustrates a typical CNN configuration featuring convolutional and pooling layers through the use of GAF-encoded images. The initial input layer comprises N×k neurons, where *k* signifies the variable count of input time series, and *N* represents the length of each univariate series. The subsequent layer involves the convolutional layer, executing convolution operations using *m* filters, convolution stride *s*, and a y×y filter size. Additionally, this layer necessitates the consideration of a non-linear transformation function *f*.

As a next step, the pooling operation is performed in which a feature map is divided into *N* equal-length segments, and then every segment is represented by its average or maximum value. After several convolution and pooling operations, the original time series is represented by a series of feature maps that connects to final output layers with *n* classes. The training of CNN is performed by a sequence of training examples: $\left(x_{1},y_{1}\right),\left(x_{2},y_{2}\right),\ldots ,\left(x_{Nsample},y_{Nsample}\right)$, with $\left(x_{t}\in R^{N\times k},y_{t}\in R^{n}\;for\;1\leq t\leq N_{sample}\right)$. The multivariate or uni-variate time series $x_{t}$ is given as input to the network, while the vector $y_{t}$ denotes the target output. The training process follows a series of steps outlined in Figure 3, culminating in the development of a highly effective CNN model.

## 4. Performance Evaluation Metric

In order to evaluate the performance of the integrated approach that combines HVACSIM+ simulated data with GAF-2DCNNs, the assessment metrics utilized comprise Precision, Recall, and F1-score (9)–(11) [32], which are defined as follows:

(9) $$Precision=\frac{TP}{\left[TP+FP\right]}\times 100\;$$

(10) $$Recall=\frac{TP}{\left[TP+FN\right]}\times 100\;$$

(11) $$F1\text{-}score=2\times \frac{Precision\times Recall}{Precision+Recall}\times 100\;$$

where Precision represents the ratio of accurately predicted positive instances to the total predicted positive instances, Recall signifies the ratio of correctly predicted positive instances to all actual positive instances, and F1-score denotes the weighted average of Precision and Recall. In these equations, TP stands for the count of true positive instances, FP represents the count of false positive instances, and FN corresponds to the count of false negative instances.

## 5. Result and Discussion

The dataset generated in Section 2 was utilized to explore the viability of the proposed GAF-2DCNNs model, depicted in Figure 3. By leveraging the data provided in Table 2 (specifically, the fifth column), an 80% random subset was used for creating the training set, leaving the remaining 20% for testing purposes. Consequently, the model was trained with 6912 samples and tested with 1728 samples. Through the utilization of 194 raw feature input datapoints, the GAF-2DCNNs methodology initiated by applying the Gramian Angular Field (GAF) technique to transform the initial time series data into image data, enabling the model to capture both temporal and spatial relationships. This transformed image data were subsequently fed into the CNNs model for the final fault type classification.

In this experiment, a sequential model was employed to construct the CNN using Keras, allowing the model to be developed layer by layer. With an input image size of [6912×64×64×1], the CNN model architecture was created, consisting of three convolutional layers, three max pooling layers, and a final fully connected classification layer. The first layer employed 32 filters, while the second and third layers utilized 64 and 128 filters, respectively. The filter size in each layer can be adjusted based on the dataset. Convolution operations employed kernel sizes of [6×6], [6×6], and [3×3] for the first, second, and third filter matrices, respectively, generating feature maps. Rectified Linear Activation (ReLU) was applied in the initial two convolutional layers prior to the max pooling step. The final classification layer utilized the Softmax activation function. For parameter optimization, the Adam (Adaptive Moment Estimation) optimizer was employed, offering an alternative to traditional stochastic gradient descent for iterative network weight updates based on training data [33]. Information regarding the number of optimized parameters in the CNN architecture for the proposed GAF-2DCNNs HVAC fault detection system is outlined in Table 3.

**Figure 4:**
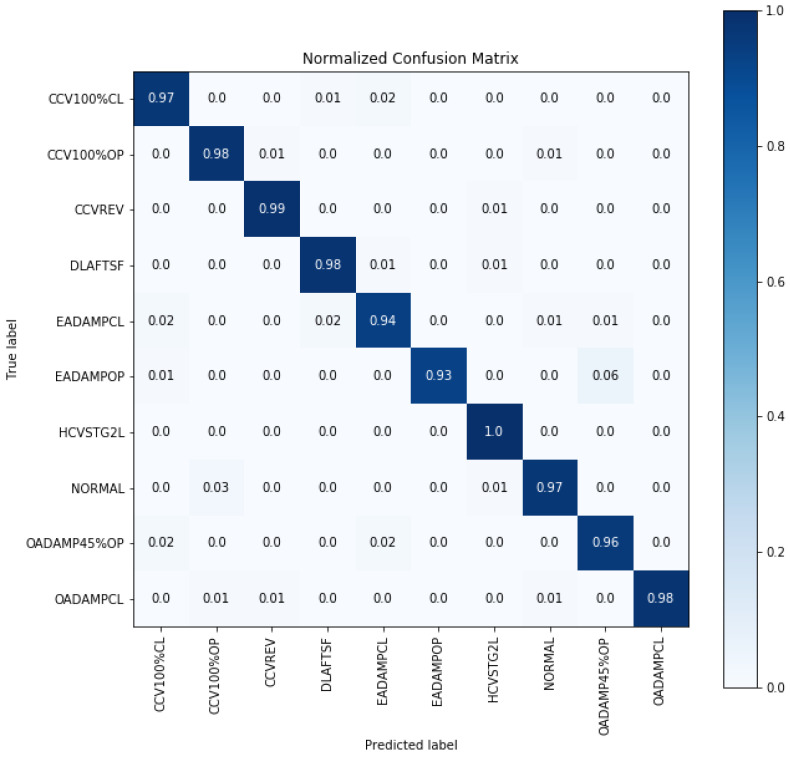
GAF-2DCNNs confusion matrix.

To assess the effectiveness of the propaosed unified framework, it is compared with other data-driven fault detection systems, including 1D-CNN, Hybrid RF-SVM, RF, and SVM, using a dataset detailed in Table 2. The dataset comprises ten classes, encompassing both normal and nine significant fault patterns associated with summer conditions. Notably, the GAF-2DCNNs demonstrates superior performance in classifying both normal and nine significant HVAC faults, achieving an better accuracy of 97%. The diagnostic accuracy is further evaluated through the utilization of a confusion matrix, as depicted in Figure 4. In the context of HVAC systems fault detection, the confusion matrix helps to visualize how well the model is performing in terms of correctly classifying different fault types, as well as identifying any misclassifications.

Upon analyzing the confusion matrix depicted in Figure 4, it is evident that the diagnostic accuracy of GAF-2DCNNs achieves 95% for most all faults and normal apart from exhaust air damper faults (EADAMPCL) with 94% accuracy, and EADAMPOP with 93% accuracy. Impressively, the proposed method achieves a remarkable 100% accuracy in detecting heating coil valve leakage faults (HCVSTG2L) and a robust 99% accuracy in identifying cooling coil valve reverse action faults (CCVREV). In HVAC systems, achieving a remarkable 100% accuracy in detecting faults such as heating coil valve leakage (HCVSTG2L) and a robust 99% accuracy in identifying cooling coil valve reverse action faults (CCVREV) signifies the exceptional ability of the system to promptly identify and diagnose these specific issues. This high accuracy level indicates that the proposed diagnostic model can effectively recognize even the slightest deviations in the behavior of heating coil valve leakage and cooling coil valve reverse action, ensuring that these faults are promptly addressed and mitigated.

Furthermore, the GAF-2DCNNs demonstrates a notable level of accuracy, specifically reaching 98% in effectively detecting cooling coil valve fully closed faults (CCV100%CL), duct leak after supply fan faults (DLAAFTSF) and outside air damper fully closed faults (OADAMPCL), as presented in Table 4. Additionally, the model shows satisfactory classification accuracy of 96% for both outside air damper partially opened faults (OADAMP45%OP) and fault-free conditions (NORMAL). While these accuracy rates showcase proficiency of the system in fault detection, it is important to note that cooling coil valve and exhaust air damper faults incur a slight misclassification rate of 1–3%. This can be attributed to the nuanced and subtle nature of symptoms associated with these faults. This level of accuracy is immensely valuable, as it enables the HVAC systems to accurately identify and address these faults, minimizing potential disruptions and optimizing overall system performance.

Besides, the primary focus of this study lies in the strategic incorporation of normal states into the analysis, presenting several advantages that clearly distinguish it from the work presented in [24]. This approach proves to be particularly valuable, x as it effectively addresses several key challenges in fault detection and diagnosis within HVAC systems. By integrating normal states into the analysis, the proposed method significantly reduces false positive outcomes. This means that the chances of incorrectly identifying a fault when the system is functioning normally are minimized, leading to more accurate and reliable fault detection. This advantage is underscored by the precision rates presented in Table 4, where precision values range from 92% to an impressive 100% across different fault categories. Such high precision rates showcases ability of the proposed method to correctly classify actual faults while maintaining a low rate of false positives.

Moreover, the emphasis on normal states enhances the accuracy of fault detection, allowing the method to better identify and categorize faults even in complex operational scenarios. This is evident from the recall rates provided in the same table, where recall values consistently surpass 94%, except for one fault category (EADAMPOP), which has a 93% recall rate. This indicates the robustness of the proposed method in capturing a high proportion of actual faults, further emphasizing its effectiveness in real-world applications. In addition, the F1-score, a metric that evaluates ability of the model to handle imbalanced datasets, highlights the proposed GAF-2DCNNs strong performance. The F1-scores provided in Table 4 demonstrate the capability to effectively manage an unbalanced input dataset, indicating its practicality in scenarios where certain fault types might be less frequent or inherently challenging to detect.

The method proposed in this study stands out for its approach to addressing model generalization, setting it apart from the approach presented in [24]. To handle this concern effectively, comprehensive testing and validation are carried out using ASHRAE data [26], which is widely recognized for its authoritative status in the heating, ventilation, and air conditioning (HVAC) field. Utilizing the same optimized GAF-2DCNNs parameters, the results demonstrate in Table 5 achieve strong generalization performance and operational stability, with an overall accuracy of 95%.

The comparison results in Table 5 highlight the remarkable performance of the proposed GAF-2DCNNs method across various significant faults when compared to both the Simulation Dataset (HVACSIM+) and the ASHRAE Dataset [26]. Notably, the GAF-2DCNNs achieves better Precision, Recall, and F1-score metrics for critical faults such as CCVREV (Precision: 99%, Recall: 99%, F1-score: 99%) and HCVSTG2L (Precision: 96%, Recall: 100%, F1-score: 98%). Equally noteworthy is its consistent and strong performance for ASHRAE-data-related faults, such as CCVREV, with a Precision of 95%, Recall of 97%, and F1-score of 97%, as well as HCVSTG2L, with a Precision of 95%, Recall of 97%, and F1-score of 97%. Similarly, other faults, including duct leak after supply fan (DLAFTSF) and exhaust air damper open (EADAMPOP) with respective Precision, Recall, and F1-scores, demonstrate acceptable performance. These results demonstrate the efficacy of the proposed method in accurate fault detection and classification across various scenarios, underlining its reliability and potential as a robust tool for HVAC fault detection in practical applications. Overall, the GAF-2DCNNs exhibit a strong performance with an impressive model accuracy of 97%, surpassing the HVACSIM+ Simulation dataset and closely aligning with the ASHRAE benchmark dataset accuracy of 95%.

Moreover, central to the distinct character of this study is a comprehensive examination of outcomes and the effectiveness of the proposed unified framework introduced here. Set apart by [24], a comprehensive assessment was undertaken by contrasting our method with recently developed machine learning techniques, encompassing 1D-CNN, Hybrid HRF-SVM, and traditional RF and SVM. While these methods demonstrate acceptable performance in classifying fault and normal states, the accuracy for specific faults remains lower than 85% in each approach, marking a significant deviation from the achieved accuracy of the proposed GAF-2DCNNs. The comparison study begins with 1D-CNN, and the experimental result in Table 6 shows that the overall accuracy of 94% is achieved with the 1D-CNN; however, some faults have a higher misclassification rate, such as the accuracy of 82% and 89% for HCVSTG2L and EADAMPCL faults.

The comparison analysis involves the evaluation of the proposed GAF-2DCNN model against recently developed machine learning techniques, including 1D-CNN, Hybrid HRF-SVM, and traditional RF and SVM. While all these methods exhibit proficient performance in classifying fault types, certain faults demonstrate classification accuracies lower than 85% within each individual method. The evaluation of 1D-CNNs, as presented in Table 6, reveals an overall accuracy of 94%, but highlights limitations in correctly identifying specific faults. Notably, the HCVSTG2L and EADAMPCL faults show higher misclassification rates (82% and 89% accuracy, respectively). These findings underscore the importance of precise fault detection, especially for HVAC operations.

The misclassification the HCVSTG2L fault may impact heating functionality, while the EADAMPCL fault can affect ventilation and indoor air quality. The trade-off between computation and accuracy is recognized, and the 3% accuracy increase showcased by GAF-2DCNN enhances FDD reliability, reinforcing the significance of incremental improvements in HVAC operations. In addition, the proposed method focuses specifically on the periods when the HVAC system is operational, and the training data are targeted to capture relevant features and patterns during the occupy period. This targeted training strategy helps optimize the computational efficiency of the GAF encoding process, as it eliminates the need to process sensor data during non-occupancy periods. As a result, the ability of the proposed approach to achieve accurate fault diagnosis is not compromised by computational burdens.

Among the recent advancements, the hybrid RF-SVM model [30] stands out for employing a raw sensor signal with 194 features with significant feature selection method, enabling a comprehensive model comparison. As shown in Table 6, this hybrid model achieves accuracy of 91% in HVAC fault detection, excelling in identifying issues such as cooling coil valve faults, outdoor air damper irregularities, exhaust air damper closures, and duct leakage. However, its overall accuracy falls short of the proposed GAF-2DCNNs model. The RF-based approach also demonstrates promise result, achieving 100% accuracy in classifying specific faults, yet it achieves an 88% overall accuracy with relatively lower performance for NORMAL and exhaust air damper opened faults compared to the GAF-2DCNNs approach.

As for the SVM-based model, the SVM-based FDD system achieves good results in exhaust air damper opened faults (EADAMPOP) and heating coil valve leakage faults (HCVSTG2L) with an accuracy of 94%. However, the accuracy in other fault type classifications is well below the accuracy of the proposed GAF-2DCNNs. The detailed comparison results of the proposed GAF-2DCNNs with other FDD systems are shown in Table 6 and it clearly indicates that the GAF-2DCNNs performs well in the fault diagnosis of HVAC systems with better accuracy of 97%. After an extensive comparison, the proposed unified framework achieves enhanced fault detection and classification accuracy in HVAC systems, surpassing existing machine learning techniques with higher accuracy.

## 6. Conclusions

Advancing HVAC fault detection methodologies, a unified framework is introduced by combining HVACSIM+ simulated data with GAF-2DCNN. The integration of Gramian Angular Field (GAF) and two-dimensional convolutional neural networks (2D-CNNs) techniques allows for the effective capture of both temporal and spatial data, enhancing fault classification accuracy for 10 major HVAC faults and normal conditions with better accuracy of 97%. The approach provides a comprehensive understanding of real-world scenarios by considering normal states during occupancy periods, emphasizing the robustness of the proposed method, achieving accurate classification between normal and faulty states, and offering meaningful predictions without requiring extra interpretability techniques. To evaluate the effectiveness of the proposed GAF-2DCNNs, a comparison is made with Support Vector Machine (SVM), Random Forest (RF), and hybrid HVAC systems fault diagnosis models. The results reveal an overall accuracy of 97%, with precision, recall, and F1 scores surpassing 90% for each fault. This underscores the robustness and effectiveness of the proposed unified framework. In summary, the improved fault detection approach seamlessly integrates HVACSIM+ simulated data, considers occupancy patterns, records data at finer 1-min intervals, and rigorously validates against established benchmarks, such as ASHRAE data. Collectively, these factors contribute to the advancement of HVAC fault detection and add robustness to the credibility of the study. While the proposed approach achieves high accuracy in HVAC fault detection, its effectiveness might still be influenced by the availability and quality of input data. In scenarios where sensor data are sparse or noisy, the model performance could be compromised. Future work could involve investigating techniques to enhance the model robustness, potentially involving data augmentation, transfer learning, or hybridization with complementary fault detection methods.

## Figures and Tables

**Table 1:** Building design and HVAC system parameters.

Description	Design Parameter
Location	Sydney, Australia
L × W of building	40 m × 40 m
Number of floor, rooms/zones	Single storey, 4
L × W of each room	20 m × 20 m
Floor to ceiling height	3.5 m
Window to floor ratio	35 %
Occupants	0.15 person/sqm
Lighting power	20 W/sqm
Equipment power	12.5 W/sqm
Shading coefficient and U value of the window	SC = 0.95, U = 6.21 W/sqmK
U value of the roof	0.795 W/sqmK
U value of the above grade wall	3.778 W/sqmK
HVAC Systems Capacity	Auto Sizing
Chiller coefficient	4.45
Chilled water temperature	7 ${}^{\circ }$C
Supply/return chilled water temperature different	5 ${}^{\circ }$C
Supply condensed water temperature	30 ${}^{\circ }$C
Supply/return condensed water temperature different	5 ${}^{\circ }$C
AHU fan power	0.000826 W/cfm
Supply air temperature set point	12.77 ${}^{\circ }$C
Zone heating and cooling point	21 ${}^{\circ }$C and 22 ${}^{\circ }$C
Control	AHU with VAV, equipped with VSD

**Table 2:** Summary of AHU faults considered in the proposed FDD model.

Fault	Abbreviation	Description	Sample
F0	NORMAL	Normal Condition	2160
F1	CCV100%OP	Control Coil Valve fully opened	720
F2	CCV100%CL	Control Coil Valve fully closed	720
F3	CCVREV	Cooling Coil Valve Reverse Action	720
F4	DLAFTSF	Duct Leaf After Supply Fan	720
F5	EADAMPOP	Exhaust Air Damper opened	720
F6	EADAMPCL	Exhaust Air Damper closed	720
F7	OADAMPCL	Outside Air Damper closed	720
F8	OADAMP45%OP	Outside Air Damper 45% opened	720
F9	HCVLSTG2	Heating Coil Valve Leak—Stg 2	720

**Table 3:** GAF-2DCNNs HVAC fault detection: CNN network architecture and parameters.

Layer	Filters	Kernel	Output	Parameters
Input Layer	-	-	(64, 64, 1)	-
Conv2D	32	6×6	(59, 59, 32)	1184
Max Pooling	-	-	(29, 29, 32)	-
Dropout	-	-	(29, 29, 32)	-
Conv2D	64	6×6	(24, 24, 64)	73,791
Max Pooling	-	-	(12, 12, 64)	-
Dropout	-	-	(12, 12, 64)	-
Conv2D	128	3×3	(10, 10, 128)	73,856
Max Pooling	-	-	(5, 5, 128)	-
Dropout	-	-	(5, 5, 128)	-
Flatten	-	-	(3200)	-
Dense	-	-	(256)	819,456
Dropout	-	-	(256)	-
Classification	-	-	(10)	2570

**Table 4:** Classification report for proposed GAF-2DCNNs.

Fault	Precision (%)	Recall (%)	F1-Score (%)
NORMAL	99	97	98
CCV100%OP	92	98	95
CCV100%CL	95	97	96
CCVREV	99	99	99
DLAFTSF	98	98	98
EADAMPOP	100	93	96
EADAMPCL	94	94	94
OADAMPCL	100	98	99
OADAMP45%OP	93	96	94
HCVSTG2L	96	100	98

**Table 5:** Comparison studies on ASHARE RP-1312 and simulated data from HVACSIM+.

Fault	Simulation Dataset (HVACSIM+)			ASHRAE Dataset [26]		
	Precision (%)	Recall (%)	F1-Score (%)	Precision (%)	Recall (%)	F1-Score (%)
NORMAL	99	97	98	98	93	95
CCV100%OP	92	98	95	97	97	97
CCV100%CL	95	97	96	86	91	91
CCVREV	99	99	99	95	97	97
DLAFTSF	98	98	98	91	93	93
EADAMPOP	100	93	96	94	95	95
EADAMPCL	94	94	94	94	87	87
OADAMPCL	100	98	99	98	97	97
OADAMP45%OP	93	96	94	93	94	94
HCVSTG2L	96	100	98	97	97	97
Model Accuracy	97			95		

**Table 6:** GAF-2DCNN evaluation with state-of-the-art FDD algorithms.

FAULT	GAF-2DCNNs (%)	1D-CNN (%)	RF-SVM (%) [30]	RF (%)	SVM (%)
NORMAL	97	96	76	73	91
CCV100%CL	97	99	94	96	69
CCV100%OP	98	98	99	97	92
CCVREV	99	95	100	100	85
DLAFTSF	98	94	97	93	73
EADAMPCL	94	89	99	100	69
EADAMPOP	93	95	90	80	94
HCVSTG2L	100	82	98	88	94
OADAMP45%OP	96	98	94	93	64
OADAMPCL	98	95	96	93	69
Model Accuracy	97	94	91	88	82

## Data Availability

The data presented in this study are available on request from the corresponding author.
